# Influence of organisational climate on public service employee physical health

**DOI:** 10.4102/hsag.v29i0.2244

**Published:** 2024-03-26

**Authors:** Bianca I. Chigbu, Willie Chinyamurindi, Chioneso S. Marange

**Affiliations:** 1 Department of Sociology, Faculty of Social Sciences and Humanities, University of Fort Hare, Alice, South Africa; 2 Department of Business Management, Faculty of Management and Commerce, University of Fort Hare, East London, South Africa; 3 Department of Statistics, Faculty of Science and Agriculture, University of Fort Hare, East London, South Africa

**Keywords:** physical health, organisational climate, decent work, South Africa, public service

## Abstract

**Background:**

The working conditions in the South African public service, notably its challenging environment, pose significant threats to the physical health of employees. Calls exist in understanding how this can be addressed.

**Aim:**

The study investigated the predictors of physical health, accounting for the role of organisational climate and decent work.

**Setting:**

The study was conducted in Bisho in the Eastern Cape province of South Africa.

**Methods:**

An instrument was administered through a survey using a sample of 289 respondents. Descriptive and inferential statistics were used to test the hypothesised relationships.

**Results:**

No significant direct effect existed to show that the sounder an organisational climate, the better the physical health of employees will be (β = –0.014, t = –0.199, *p* = 0.843, 95% confidence interval [CI] [–0.153 to 0.125]). However, statistically significant evidence existed to show that the more focus on promoting decent work, the better the physical health of employees will be (β = 0.463, *p* = < 0.001, 95% CI [0.258 to 0.668]). Finally, decent work has a full mediating effect on the relationship between organisational climate and employee physical health (β = 0.105, 95% CI [0.054 to 0.167]).

**Conclusion:**

Public service organisations need to pay attention to the role of its climate and decent working conditions in promoting employee physical health.

**Contribution:**

Interventions are needed centered on improving decent work and the organisational climate as identified predictors of employee physical health.

## Introduction

The working conditions in an organisation can significantly impact the health of employees (Shava & Chinyamurindi 2021). The organisational climate comprises how work is organised and the responses that accompany all this from a leadership perspective (Chinyamurindi, Mathibe & Marange 2023). Such an organisational climate is potentially weighted with employee and organisational outcomes (Wang et al. 2021). A healthy organisational climate can affect employees’ physical health (Duffy et al. 2021; Godderis & Lambrechts 2019). Physical health is the overall well-being and functioning of an individual’s body systems and organs (Wang et al. 2021). Physical health encompasses aspects such as cardiovascular health, muscular strength, flexibility, respiratory function, and overall fitness (Karthick et al. 2022; Morsi et al. 2017; Pega et al. 2021). An employee’s sense of well-being is affected by a range of factors. These include organisational context and the scope of activities an employee engages in (International Labour Organization 2009; Godderis & Lambrechts 2019). These factors highlight the importance of the organisational climate, as it plays a crucial role in shaping the working conditions and the overall well-being of employees (Sanhokwe, Chinyamurindi & Muzurura 2023). Calls exist for more research that seeks to promote a work environment that supports decent working conditions (Kabir, Gunu & Gwadabe 2022; Kekana, Koekemoer & O’Neil 2022). An environment promoting decent working conditions is linked to better physical health (Duffy et al. 2021) and the promotion of mental well-being (Adams 2019; Chinyamurindi et al. 2023).

Calls exist for the prioritisation of physical health, especially within the workplace (International Labour Organization 2009). The focus in promoting physical health is magnified by the guidelines set by the World Health Organization on physical activity and sedentary behaviour (World Health Organization 2020). The promotion of such physical health must be equalled with the existence of a conducive workplace (Lukan et al. 2022). Thus, access to support services that support the development of well-being within organisations becomes crucial (Busch et al. 2022). Most organisational behaviour studies have given scant focus to physical health issues within the workplace (Mathibe & Chinyamurindi 2021), rather than mental health. Further, the effect of organisational climate factors on employee physical health is also an issue that has received scant limited empirical focus (Chinyamurindi & Shava 2022). This under-explored gap in the literature provided the foundational rationale for this research study.

The study is in the context of the South African public service. According to Fihla and Chinyamurindi (2018), the public service is an essential vehicle through which service delivery outcomes are realised. Yet in doing so the same public service becomes a stressful context to work in, with a potential impact on the health of employees (Shava & Chinyamurindi 2021). A need exists for targeted interventions that assist the promotion of health especially within the public service (Chinyamurindi & Shava 2022; Mathibe & Chinyamurindi 2021; Shava & Chinyamurindi 2021). Such targeted interventions can potentially assist employees to work better in meeting their work outcomes (Ruzungunde, Chinyamurindi & Marange 2023).

Although decent work is a crucial subset of organisational climate, it consists of distinct components such as fair compensation and job security. By considering each component separately, their distinct effects on the well-being of employees may be examined (Sanhokwe et al 2023). In addition, the intricate interrelationships between these components demand separate research to comprehend how elements such as fair salaries influence job satisfaction in the context of the larger company culture (Chinyamurindi & Shava 2022). Moreover, distinct organisational contexts prioritise distinct components of decent employment, necessitating individualised solutions. By promoting aspects related to decent working conditions, this has the potential to also enhance the organisational climate. Specific policy implications related to decent work differ from those involved with more significant organisational climate initiatives, necessitating a separate examination in order to provide meaningful policy suggestions (Chinyamurindi & Shava 2022).

Therefore, the aim of this study on public sector employees was to investigate the relationship between a more sounder organisational climate and better physical health of employees through strengthening decent work.

Organisational climate emerges as a significant determinant of the well-being of public sector employees, although decent working circumstances play an equally important role in this dynamic. A thorough analysis was required to appreciate the intricate relationship between these two factors and their effect on physical health. An in-depth analysis could provide priceless insights that will serve as the essential foundation for policy formulation and focused action.

This study conducted in the public sector was not only to confirm but also to enhance the insight of how organisational climate and decent work influence employee physical health in this particular organisational context. This research has the potential to inform public sector- and context-specific activities and policies. Employees can benefit through streamlined interventions that not only enhance aspects of decent work but also their physical health. For managers in the public sector, findings from the study can be useful in encouraging a productive work context.

## Literature

To gain an understanding of the secondary data relevant to the research aim, the literature review considered the main constructs of organisational climate, decent work, and employee physical health. The following section builds the conceptualisation leading to the formulated hypotheses.

### Theoretical literature

This study focused on two theories. Firstly, centred on the role of organisational climate, the organisational climate theory proposed by Renis Likert in the 1960s was considered. The logic here is that the existence of an organisational environment that is conducive to work may realise positive health outcomes (Schneider, González-Romá, Ostroff & West 2017). In essence, the organisational climate is viewed as consisting of the policies, procedures, people, finance, and practices within the organisation (Schneider, Wheeler & Cox 1992).

Secondly, the Psychology of Working Theory (PWT) (Duffy et al. 2016) was considered. The PWT covers aspects related to decent work as having an essential role in the lives of working adults (Blustein 2001). Such quests for decent work can be linked to positive workplace behaviours and health outcomes (Duffy et al. 2021). The tenets of decent work include: (1) physically and interpersonally safe working conditions, (2) access to healthcare, (3) adequate compensation, (4) hours that allow free time and rest, and finally, (5) organisational values that complement family and social values (Duffy et al. 2016).

In theoretical terms, organisational climate can be perceived as a micro-level construct, focusing on the internal dynamics and psychological atmosphere within a specific organisation. The organisational climate encompasses factors such as leadership styles, communication patterns, and overall employee satisfaction (Moslehpour et al. 2019; Ratnasari, Sutjahjor & Adam 2019; Sadiartha & Sitorus 2018).

On the other hand, the concept of decent work operates at a macro-level, transcending individual organisations and addressing global socio-economic rights and the overall well-being of workers. The goal of decent work is to establish a universally fair and equitable working world. While a positive organisational climate contributes significantly to creating a conducive work environment (Polo Escobar et al. 2022; Zulfqar, Liaqat & Nazir 2012), decent work encompasses a broader spectrum of labour rights and social protections (Ghai 2003), ensuring not only a harmonious workplace atmosphere but also fundamental aspects such as fair wages, job security, and dignified working conditions (Del Rocio Garcia Sanchez et al. 2022).

### Empirical literature

Organisational climate, a multifaceted term, encompasses an organisation’s structure and activities, constituting the context in which employees perform their duties (Mohammadi & Youzbashi 2012). This climate includes various elements such as the support given to employees, workload arrangements, and how employees adapt to their work context (Massoudi & Hamdi 2017; Lehto et al. 2020). A dynamic environment is established that sets the stage for the vital connection between organisational climate and the concept of decent working conditions, enabling employees to thrive (Kekana et al. 2022). A pivotal factor in ensuring decent working conditions is the presence of a safe working environment that promotes comfort and ease for employees (Zondo 2021). A workspace free from impediments fosters physical well-being and contributes to a conducive organisational climate, facilitating productive work expression (Di Fabio & Kenny 2019; Fabry, Broeck & Maertens 2022; Vignoli et al. 2020). Trust, skills development opportunities, and the promotion of physical health issues further bolster a positive organisational climate (Chigbu & Nekhwevha 2020, 2021; Kekana et al. 2022; Rossier & Oudraogo 2021).

Promoting employee physical health not only enhances well-being but also fuels professional development (Lehto et al. 2020). Professional growth necessitates the unwavering support of management, ensuring the work environment is free from health hazards (Kabir et al. 2022). Such support positively impacts employees’ psychological well-being, fostering feelings of worthiness and dignity (Rossier & Ouedraogo 2021). Crucially, the organisational climate must be intricately linked with psychological support for employees, establishing a crucial connection between physical and psychological well-being (Ribeiro et al. 2018; Vignoli et al. 2020). This integration is facilitated through organisational processes and activities (Yang, Jiang & Pu 2021). Studies consistently emphasise the detrimental consequences of an organisational climate that fails to promote decent work, often leading to unacceptable working conditions and adversely affecting employees’ physical and mental health (d’Errico et al. 2022; Garcia-Lozano et al. 2022; Kaya-Aytutuldu, Birinci & Tarakcı 2022; Yang et al. 2021). A conducive organisational climate is indispensable, encouraging the physical health and holistic well-being of employees (Viitala, Tanskanen & Santtl 2015).

Addressing issues related to work methods and tools forms a pragmatic starting point (Basakci Calik et al. 2020). This initial approach necessitates a focus on the physical aspects of employee engagement in the public sector, associated with work is imperative, ensuring prompt resolution to prevent potential long-term health effects (Besharati et al. 2020; d’Errico et al. 2022; Kaya-Aytutuldu et al. 2022). It can be assumed that organisations can establish a work environment that fosters both physical and psychological well-being, thereby creating an environment conducive to decent work. Based on the presented literature, the following hypotheses were set for this study:

**Alternative Hypothesis (H_a_) 1:** *The sounder an organisational climate, the better the physical health of employees will be.*

**Alternative Hypothesis (H_a_) 2:** *The more focus on promoting decent work, the better the physical health of employees will be.*

**Alternative Hypothesis (H_a_) 3:** *The relationship between a sounder organisational climate and better physical health of employees will be strengthened by more focus on promoting decent work.*

**FIGURE 1:**
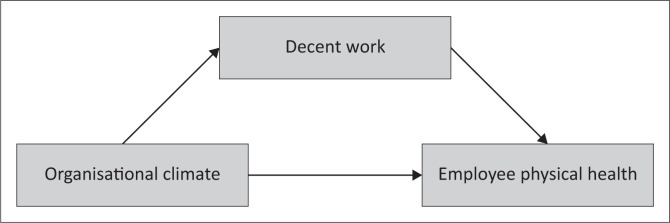
Conceptual framework for the study.

Figure 1 presents the research framework for the study.

## Research methods and design

### Measures

The survey relied on using existing instruments that are readily available online to share and use. To measure organisational climate, an instrument developed by Peña-Suárez et al. (2013) was used with a total of 35 items. To measure decent work, an instrument by Duffy and Colleagues was used (Duffy et al. 2017) with a total of 15 items. Finally, to measure physical health, an instrument developed by Cleeland and Ryan (1994) was used with a total of six items. All the items of the three tools were measured on a 5-point Likert scale. Concerning reliability, the scales all complied with sufficient ratings of acceptable Cronbach’s alpha values of 0.7, as set by Nunnally (1978) and to be shown in the Results section.

### Respondents

A cross-sectional survey was utilised with government employees in the Eastern Cape province of South Africa. The sampled group (*n* = 289) was obtained through a convenience sampling approach. Data were collected from the Departments of Social Development, Department of Education, and from the Department of Public Works. These departments were conveniently accessed as they indicated interest in taking part in the research.

### Ethical considerations

The research team applied for ethical clearance through the participating institution, University of Fort Hare. From this process, an institutional ethical clearance certificate was issued (Reference number: CHI1028).

### Data-analysis procedure

The Statistical Package for the Social Sciences (SPSS) version 27 and the Hayes process macro for SPSS (Hayes 2013) were used for the data analysis. Before running the analysis, the authors examined the main theoretical variables to determine if ordinary least squares regression (OLS) based path analysis for the Hayes process macro was appropriate. Thus, the assumptions for linearity, homoscedasticity, normality, and independence of observations had to be assessed for mediation analysis to be possible. For linearity, the relationship between the independent variable, organisational climate and the dependent variable, employee physical health should be linear to minimise error (Hayes 2013). To assess this assumption, residuals against predicted values were plotted in the linear regression models for organisational climate and decent work in predicting employee physical health.

For homoscedasticity, it is required that the estimation error be relatively equal across all predicted values of the dependent variable (Hayes 2013). To check this assumption, the same plots for linearity were examined but this time assessing the consistency if the data spread consistently in the plot which should resemble a rectangle. Further, to examine normality, Q-Q plots were used, and normality was achieved once the data points are seen to consistently lie on the diagonal line. Given the fact that the authors randomly sampled the respondents, it is unlikely that there are underlying issues that would compromise the independence of the collected data. After verifying that all these assumptions were met, the Cronbach’s alpha coefficient was used to assess the reliability of the study’s theoretical variables and constructs.

According to Nunnally and Bernstein (1994), Cronbach’s alpha values of 0.70 and higher are satisfactory. For hypothesis testing, OLS regression-based path analysis using the Hayes process macro in SPSS was used. Following MacKinnon et al. (2004), the strength of the direct and the indirect effects were used to determine the result of the hypothesised frameworks. Concerning the alternative hypotheses 1 and 2, the significance of these hypothesised frameworks (direct linear relationships) was assessed using the direct effects of the OLS regression beta path estimates. For the third alternative hypothesis, the significance of the mediating effect (indirect effect) of decent work was examined using 95% bias-corrected confidence intervals based on 10 000 bootstrap samples.

## Results

Based on the results for the internal consistency (see Table 1), Cronbach’s alpha values for the instruments show acceptable reliability coefficients. Thus, organisational climate had an overall Cronbach’s alpha coefficient of 0.909, employee physical health reported a Cronbach’s alpha coefficient of 0.770, and decent work reported a Cronbach’s alpha coefficient of 0.741. The reported Cronbach’s alpha coefficients for the different dimensions of decent work (all Cronbach’s alpha values > 0.70) are also considered acceptable. Table 1 also shows the descriptive statistics of the established theoretical variables and constructs. Measured on 5-point Likert scales, all variables and constructs reported moderate mean levels with free time and rest (Mean = 4.14; standard deviation [s.d.] = 0.85) having a moderately high mean rating among the public service employees.

**TABLE 1:** Descriptive and reliability analysis (*N* = 289).

Variables and constructs	Mean	s.d.	Number of Items used	Cronbach’s α
Organisational climate	2.99	0.59	35	0.909*
Employee physical health	3.14	0.69	6	0.770*
Decent work	3.33	0.40	15	0.741*
1. Safe working conditions	3.28	0.70	3	0.763*
2. Access to healthcare	3.32	0.75	3	0.749*
3. Adequate compensation	3.01	0.51	3	0.798*
4. Free time and rest	4.14	0.85	3	0.796*
5. Complementary values	2.90	0.77	3	0.869*

Note: Organisational climate and decent work were measured on a 5-point Likert scale (1 – Strongly Disagree, 2 – Disagree, 3 – Neutral, 4 – Agree, and 5 – Strongly Agree). Employee physical health was measured on a 5-point Likert scale (1 – None of the time, 2 – A little of the time, 3 – Some of the time, 4 – Most of the time, 5 – All the time).

s.d., standard deviation.

* , Significantly acceptable reliability.

Table 2 shows the results of the direct and indirect beta coefficients for the OLS regression-based path analysis using the Hayes process macro.

**TABLE 2:** Direct and indirect beta coefficients for the mediating role of decent work on the relationship between organisational climate and employee physical health.

Effects	Unstandardised beta coefficients		Significance of beta coefficients		95% confidence interval	
	beta	s.e.	*t*-value	*p*-value	LLCI	ULCI
**Direct effect(s)**						
a) OC (X) → DW (M)	0.227*	0.038	6.031	< 0.001	0.153	0.301
b) DW (M) → PH (Y)	0.463*	0.104	4.454	< 0.001	0.258	0.668
c) OC (X) → PH (Y)	−0.014	0.070	−0.199	0.843	−0.153	0.125
**Total effect of X on Y**	0.091	0.068	1.332	0.184	−0.044	0.226
**Indirect effect of X on Y**	0.105*	0.029	-	-	0.054	0.167

Note: Number of bootstrap samples for percentile bootstrap confidence intervals: 10 000; Independent variable (X): Organisational Climate (OC); Dependent variable (Y): Employee Physical Health (PH); Mediator variable (M): Decent Work (DW).

s.e., standard error; LLCI, lower limit confidence interval; ULCI, upper limit confidence interval.

* , Significant effect at alpha = 0.05.

**FIGURE 2:**
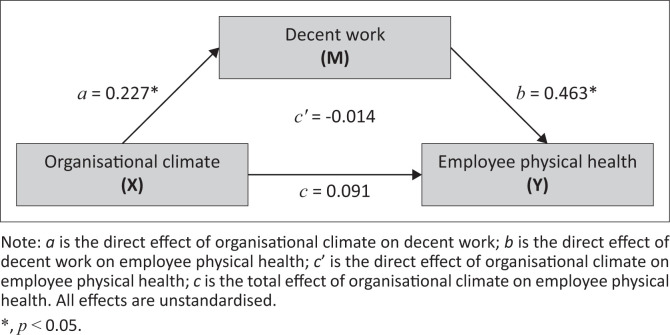
Regression beta path coefficients for the conceptual model.

Alternative hypothesis (H_a_) 1 proposed that the sounder an organisational climate, the better the physical health of employees will be. From Table 2 and Figure 2, results from the regression beta path coefficients indicated that organisational climate had no significant predictive effect on the physical health of employees (β = –0.014, *t* = –0.199, *p* = 0.843, 95% CI [–0.153 to 0.125]).

This result does not support alternative hypothesis (H_a_) 1. Thus, there exists insufficient evidence to conclude that there is a significant direct and positive linear relationship between organisational climate and the physical health of employees. Therefore, from the hypothesised conceptual model, sounder organisational climate does not lead to better physical health of employees within the public service sector.

Alternative hypothesis (H_a_) 2 proposed that the more the focus on promoting decent work, the better the physical health of employees will be. Examining the beta path coefficient of the direct effect of decent work on the physical health of employees, the results in Table 2 and Figure 2 fully support alternative hypothesis (H_a_) 2 (β = 0.463, *t* = 4.454, *p* ≤ 0.001, 95% CI [0.258–0.668]). As the beta path coefficient is positive and statistically significant, this indicates a significant and direct linear relationship between promoting decent work and the physical health of employees. Thus, it can be concluded that the more the focus on promoting decent work, the better the physical health of employees will be in the public service sector.

To determine the mediating effect of decent work, the 95% bias-corrected confidence intervals (see Table 2) indicate that the indirect effect of decent work on the relationship between organisational climate and the physical health of employees is positive and statistically significant (β = 0.105, 95% CI [0.054–0.167]). As the direct effect of organisational climate on the physical health of employees was reported not to be statistically significant (see result in [H_a_] 1), this results in support of full mediation. The findings show support for the relationship between a sounder organisational climate to be linked with the better physical health of employees strengthened by a focus on promoting decent work. In conclusion, decent work has a full mediating effect or role on the relationship between organisational climate and employee physical health in the public service sector.

## Discussion

The study was aimed at investigating the predictors of physical health accounting for the role of organisational climate and decent work among public service employees in South Africa. The findings of the study show support for the idea that working conditions have an impact on the health of employees (Duffy et al. 2021; Godderis & Lambrechts 2019). Decent work becomes important in promoting employee physical health. The promotion of a working environment that supports decent work potentially assists employees not only in how they do their jobs but also with their work outcomes (Kabir et al. 2022; Kekana et al. 2022). In promoting decent working conditions, organisations may also potentially help in promoting aspects of physical health (Duffy et al. 2021). Findings in this study contribute to the literature on health development within the South African public service; notably, focus has been chiefly centred on employee mental health (Mathibe & Chinyamurindi 2021; Shava & Chinyamurindi 2021). Physical health promotion is argued for in this study.

There is a need for the promotion of physical health within organisations such as the public service. Leaders and managers within the public service would do their best to be familiar with the guidelines on aspects related to the promotion of physical health (World Health Organization 2020). Efforts like this, as advocated in the literature, should be equalled by a conducive workplace (Lukan et al. 2022). This study shows the importance of decent work as an essential condition in the promotion of employee physical health. This finding becomes important in the South African public service noted to be with challenges (Fihla & Chinyamurindi 2018). The results magnify the role of organisational climate and decent work and the effect on employee physical health. This potentially extends focus on this aspect of health, given the noted attention to employee mental health (Mathibe & Chinyamurindi 2021; Shava & Chinyamurindi 2021).

### Implications

Some implications can be drawn. Firstly, considering the theories used (Organisational Climate Theory and Psychology of Working Theory), the study shows support for these theories as helpful in understanding organisational climate and decent work (Duffy et al. 2016). Secondly, some practical implications can be drawn from the study. Organisations may devote time during work hours for employees to be engaged in some form of activities that promote their health. Another consideration is to address those aspects related to decent work (Duffy et al. 2017). This may include making sure that a safe physical working environment exists. Such a working environment can be a valuable precursor to the promotion of employee physical health. Finally, policymakers can integrate aspects related to the guidelines on physical health and behaviour within practices of the public service. These guidelines as international imperatives (World Health Organization 2020) should translate to the public service as a context of work. The role of managers, leaders, and policymakers can be a helpful vehicle for this journey.

## Conclusion

At a time when the drive is towards the promotion of employee mental health in organisations, efforts towards physical health should not be neglected. A starting point could be to give these aspects equal priority in the contemporary workplace. Further, an integrative role in efforts towards the promotion of employee health is argued for. Ultimately, two issues are focal. Firstly, the productivity of the workplace through paying attention to the needs of employees, especially their health. Secondly, the necessity for organisational interventions that promote a conducive organisational context for work while promoting decent working conditions.

### Limitation and recommendations

Some limitations can be drawn from the study. First of all, due caution should be exercised as the findings are not generalisable outside the scope of the respondents. In this case, respondents comprised working adults within the South African public service. Future studies could replicate this study in different national and work contexts as a means of comparative studies. Other important organisational characteristics and variables could be measured against the physical health aspect explored as an outcome variable in this study. However, the study does proffer some valuable theoretical and practitioner insights. The study identifies those factors within the organisation as measured by organisational climate and how this influences employee physical health. This can become a valuable premise for interventions that encourage health aspects such as physical health, especially in demanding work conditions as those experienced in the South African public service. In so doing, the study does answer calls for such inquiry through the lens of the workplace (Valenzuela, Flinchbaugh & Rogers 2020).

## References

[CIT0001] Adams, J., 2019, ‘The value of worker well-being’, *Public Health Reports* 134(6), 583–586. 10.1177/0033354919878434 31600480 PMC6832080

[CIT0002] Basakci Calik, B., Yagci, N., Oztop, M. & Caglar, D., 2022, ‘Effects of risk factors related to computer use on musculoskeletal pain in office workers’, *International Journal of Occupational Safety and Ergonomics* 28(1), 269–274. 10.1080/10803548.2020.1765112 32374214

[CIT0003] Besharati, A., Daneshmandi, H., Zareh, K., Fakherpour, A. & Zoaktafi, M., 2020, ‘Work-related musculoskeletal problems and associated factors among office workers’, *International Journal of Occupational Safety and Ergonomics* 26(3), 632–638. 10.1080/10803548.2018.1501238 30015596

[CIT0004] Blustein, D.L., 2001, ‘Extending the reach of vocational psychology: Toward an inclusive and integrative psychology of working’, *Journal of Vocational Behaviour* 59(2), 171–182. 10.1006/jvbe.2001.1823

[CIT0005] Busch, S.H., Tomaino, M., Netwton, H. & Meara, E., 2022, ‘Access to mental health support services in accountable care organisations: A national survey’, *Healthcare* 10(1), 1–16. 10.1016/j.hjdsi.2022.100613 PMC8944208 35081475

[CIT0006] Chigbu, B.I. & Nekhwevha, F.H., 2021, ‘The future of work and uncertain labour alternatives as we live through the industrial age of possible singularity: Evidence from South Africa’, *Technology in Society* 67, 101715. 10.1016/j.techsoc.2021.101715

[CIT0007] Chigbu, B.I. & Nekhwevha, F.H., 2022, ‘The extent of job automation in the automobile sector in South Africa’, *Economic and Industrial Democracy* 43(2), 726–747. 10.1177/0143831X20940779

[CIT0008] Chinyamurindi, W., Mathibe, M. & Marange, C.S., 2023, ‘Promoting talent through managing mental health: The role of decent work and organisational citizenship behaviour’, *South African Journal of Industrial Psychology* 49, a2057. 10.4102/sajip.v49i0.2057

[CIT0009] Chinyamurindi, W.T. & Shava, H., 2022, ‘Determinants of employee physical and mental health: The role of career adaptability and workplace attachment in the South African Public Service’, *African Journal of Employee Relations* 45, 1–22. 10.25159/2664-3731/9015

[CIT0010] Cleeland, C.S. & Ryan, K.M., 1994, ‘Pain assessment: The global use of the Brief Pain Inventory’, *Annals Academy of Medicine Singapore* 23(2), 129–138. 8080219

[CIT0011] Del Rocio Garcia Sanchez, Dra. M., Ascencio Lopez, N., Godinez Alarcon, G., Reyes Anorve, Dr. J., Hernandez Vinalay, Dra. K., & Mayren Rodriguez Herrera, Mtra. V. 2022, ‘Labor Precariousness and its Prevalence in Students of the Law and Psychology Schools of the Autonomous University of Guerrero, Mexico as a Vulnerable Sector,’ *International Journal of Advanced Research* 10(3), 420–433. 10.21474/ijar01/14405

[CIT0012] d’Errico, A., Ardito, C., Leombruni, R., Ricceri, F., Costa, G., Sacerdote, C. et al., 2022, ‘Working conditions and health among Italian ageing workers’, *Social Indicators Research* 162, 1043–1067. 10.1007/s11205-021-02862-w

[CIT0013] Di Fabio, A. & Kenny, M.E., 2019, ‘Decent work in Italy: Context, conceptualization, and assessment’, *Journal of Vocational Behavior* 110(Part A), 131–143. 10.1016/j.jvb.2018.10.014

[CIT0014] Duffy, R.D., Allan, B.A., England, J.W., Blustein, D.L., Autin, K.L., Douglass, R.P. et al., 2017, ‘The development and initial validation of the Decent Work Scale’, *Journal of Counseling Psychology* 64(2), 206–222. https://psycnet.apa.org/doi/10.1037/cou0000191 28165257 10.1037/cou0000191

[CIT0015] Duffy, R.D., Blustein, D.L., Diemer, M.A. & Autin, K.L., 2016, ‘The psychology of working theory’, *Journal of Counseling Psychology* 63(2), 127. 10.1037/cou0000140 26937788

[CIT0016] Duffy, R.D., Prieto, C.G., Kim, H.J., Raque-Bogdan, T.L. & Duffy, N.O., 2021, ‘Decent work and physical health: A multi-wave investigation’, *Journal of Vocational Behavior* 127, 10.1016/j.jvb.2021.103544

[CIT0017] Fabry, A., Broeck, G.V.d. & Maertens, M., 2022, ‘Decent work in global food value chains: Evidence from Senegal’, *World Development* 152, 1–18. 10.1016/j.worlddev.2021.105790

[CIT0018] Fihla, S. & Chinyamurindi, W.T., 2018, ‘Human resources management practices on employee commitment: The case of a local municipality in South Africa’, *Journal of Public Administration* 53(2), 215–233, viewed 12 May 2019, from https://hdl.handle.net/10520/EJC-1328c41170.

[CIT0019] Garcia Lozano, A.J., Decker Sparks, J.L., Durgana, D.P., Farthing, C.M., Fitzpatrick, J., Krough-Poulsen, B. et al., 2022, ‘Decent work in fisheries: Current trends and key considerations for future research and policy’, *Marine Policy* 136, 104922. 10.1016/j.marpol.2021.104922

[CIT0020] Ghai, D., 2003, ‘Decent work: Concept and indicators’, *International Labour Law Review* 142(2), 113–145, 10.1111/j.1564-913X.2003.tb00256.x

[CIT0021] Godderis, L. & Lambrechts, M.C., 2019, *Make healthy employees a priority and prevent chronic diseases*, International Labour Organization, Switzerland.

[CIT0022] Hayes, A.F., 2013, *Introduction to mediation, moderation, and conditional process analysis: A regression-based approach*, Guilford Press, New York, NY.

[CIT0023] International Labour Organization, 2009, *Workplace well-being*, viewed 13 June 2023, from https://www.ilo.org/safework/areasofwork/workplace-health-promotion-and-well-being/WCMS_118396/lang--en/index.htm.

[CIT0024] Kabir, I., Gunu, U. & Gwadabe, Z.L., 2022, ‘Decent work environment and work-life balance: Empirical analysis of banking sector of hostile environments’, *Journal of Family and Economic Issues* 44, 297–312. 10.1007/s10834-022-09843-2

[CIT0025] Karthick, S., Kermanshachi, S., Asce, M. & Namian, M., 2022, ‘Physical, mental, and emotional health of construction field labors working in extreme weather conditions: Challenges and overcoming strategies, in F. Jazizadeh, T. Shealy & M.J. Garvin (eds.), Proceedings of the ASCE Construction Research Congress (CRC), Arlington, Virginia, March 9–12, 2022.

[CIT0026] Kaya-Aytutuldu, G., Birinci, T. & Tarakcı, E., 2022, ‘Musculoskeletal pain and its relation to individual and work-related factors: A cross-sectional study among Turkish office workers who work using computers’, *International Journal of Occupational Safety and Ergonomics* 28(2), 790–797. 10.1080/10803548.2020.1827528 32965164

[CIT0027] Kekana, E., Koekemoer, E. & O’Neil, S., 2022, ‘Unpacking the concept of decent work in the psychology of working theory for Blue-Collar Workers’, *Journal of Career Development* 50(2), 1–22. 10.1177/08948453221086980

[CIT0028] Lehto, R.H., Heeter, C., Forman, J., Shanafelt, T., Kamal, A., Miller, P. et al., 2020, ‘Hospice employees’ perceptions of their work environment: A focus group perspective’, *International Journal of Environmental Research and Public Health* 17(17), 1–16. 10.3390/ijerph17176147 PMC7503310 32847036

[CIT0029] Lukan, J., Bolliger, L., Pauwels, N.S., Luštrek, M., Bacquer, D.D. & Clays, E., 2022, ‘Work environment risk factors causing day-to-day stress in occupational settings: A systematic review’, *BMC Public Health* 22(1), 1–13. 10.1186/s12889-021-12354-8 35123449 PMC8818147

[CIT0030] Mathibe, M.S. & Chinyamurindi, W.T. 2021, ‘Determinants of employee mental health in the South African public service: The role of organizational citizenship behaviours and workplace social support’, *Advances in Mental Health* 19(3), 306–317. 10.1080/18387357.2021.1938153

[CIT0031] Massoudi, A.H. & Hamdi, S.S.A., 2017, ‘The consequence of work environment on employees productivity’, *IOSR Journal of Business and Management* 19(1), 35–42. 10.9790/487X-1901033542

[CIT0032] MacKinnon, D.P., Lockwood, C.M. & Williams, J., 2004, ‘Confidence limits for the indirect effect: Distribution of the product and resampling methods’, *Multivariate Behavioral Research* 39, 99–128. 20157642 10.1207/s15327906mbr3901_4 PMC2821115

[CIT0033] Mohammadi, A. & Youzbashi, A., 2012, ‘Investigation relationship of schools organization climate with job stress among physical education teachers’, *Procedia – Social and Behavioral Sciences* 47, 138–140. 10.1016/j.sbspro.2012.06.627

[CIT0034] Morsi, R.Z., Safa, R., Baroud, S.F., Fawaz, C.N., Farha, J.I., El-Jardali, F. et al., 2017, ‘The protracted waste crisis and physical health of workers in Beirut: A comparative cross-sectional study’, *Environmental Health: A Global Access Science Source* 16(1), 39. 10.1186/s12940-017-0240-6 28399867 PMC5387191

[CIT0035] Moslehpour, M., Altantsetseg, P., Mou, W. & Wong, W.K., 2019, ‘Organizational climate and work style: The missing links for sustainability of leadership and satisfied employees’, *Sustainability* 11(1), 1–17. 10.3390/su11010125

[CIT0036] Nunnally, J., 1978, *Psychometric theory*, 2nd edn., McGraw-Hill, New York, NY.

[CIT0037] Nunnally, J.C. & Bernstein, I.H., 1994, *Psychometric theory*, McGraw-Hill, New York, NY.

[CIT0038] Pega, F., Náfrádi, B., Momen, N.C., Ujita, Y., Streicher, K.N., Prüss-Üstün, A.M. et al., 2021, ‘Global, regional, and national burdens of ischemic heart disease and stroke attributable to exposure to long working hours for 194 countries, 2000–2016: A systematic analysis from the WHO/ILO joint estimates of the work-related burden of disease and injury’, *Environment International* 154, 106595. 10.1016/j.envint.2021.106595 34011457 PMC8204267

[CIT0039] Peña-Suárez, E., Muñiz, J., Campillo-Álvarez, A., Fonseca-Pedrero, E. & García-Cueto, E., 2013, ‘Assessing organizational climate: Psychometric properties of the CLIOR scale’, *Psicothema* 25(1), 137–144, viewed 13 July 2023, from https://www.psicothema.com/pdf/4092.pdf. 23336556 10.7334/psicothema2012.260

[CIT0040] Polo Escobar, B.R., Hinojosa Salazar, C.A., Sandoval Caicedo, J.H. & Castaneda Sanchez, W.A., 2022, ‘Work climate as a determining factor in organizational commitment’, *Universidad Ciencia y Tecnología* 26(114), 60–71. 10.47460/uct.v26i114.591

[CIT0041] Ratnasari, S.L., Sutjahjor, G. & Adam, 2019, ‘Employees’ performance: Organizational culture and leadership style through job satisfaction’, *Humanities and Social Sciences Reviews* 7(5), 597–608. 10.18510/hssr.2019.7569

[CIT0042] Ribeiro, R.P., Marziale, M.H.P., Martins, J.T., Galdino, M.J.Q. & Ribeiro, P.H.V., 2018, ‘Occupational stress among health workers of a university hospital’, *Revista gaucha de enfermagem* 39, 1–6. 10.1590/1983-1447.2018.65127 30043951

[CIT0043] Rossier, J. & Ouedraogo, A., 2021, ‘Work volition, decent work, and work fulfilment, in the formal and informal economy in Burkina Faso’, *British Journal of Guidance and Counselling* 49(2), 255–271. 10.1080/03069885.2021.1879991

[CIT0044] Ruzungunde, V., Chinyamurindi, W.T. & Marange, C.S., 2023, ‘Determinants of mental health: Role of organisational climate and decent work amongst employees’, *South African Journal of Human Resource Management* 21, a2105. 10.4102/sajhrm.v21i0.2105

[CIT0045] Sadiartha, A.A.N.G. & Sitorus, S.A., 2018, ‘Organizational culture, communication and leadership style on job satisfaction’, *International Journal of Research in Business and Social Science* 7(4), 1–9. 10.20525/ijrbs.v7i4.889

[CIT0046] Sanhokwe, H., Chinyamurindi, W.T. & Muzurura, J., 2023, ‘Decent work and innovative work behaviour: The mediating roles of organisational learning and work engagement’, *International Journal of Innovation Management* 27(03n04), 2350021. 10.1142/S1363919623500214

[CIT0047] Schneider, B., González-Romá, V., Ostroff, C. & West, M.A., 2017, ‘Organizational climate and culture: Reflections on the history of the constructs in the Journal of Applied Psychology’, *Journal of Applied Psychology* 102(3), 468–482. 10.1037/apl0000090 28125256

[CIT0048] Schneider, B., Wheeler, J. & Cox, J., 1992, ‘A passion for service using content analysis to explicate service climate themes’, *Journal of Applied Psychology*, 77(5), 705–716. 10.1037/0021-9010.77.5.705

[CIT0049] Shava, H. & Chinyamurindi, W.T., 2021, ‘The moderating role of career adaptability on the relationship between workplace spirituality and employee mental and physical health’, *South African Journal of Human Resource* 19, 1–11. 10.4102/sajhrm.v19i0.1437

[CIT0050] Valenzuela, M.A., Flinchbaugh, C. & Rogers, S.E., 2020, ‘Can organizations help adjust?: The effect of perceived organizational climate on immigrants’ acculturation and consequent effect on perceived fit’, *Journal of International Management* 26(3), 100775. 10.1016/j.intman.2020.100775

[CIT0051] Viitala, R., Tanskanen, J. & Säntti, R., 2015, ‘The connection between organizational climate and well-being at work’, *International Journal of Organizational Analysis* 23(4), 606–620. 10.1108/IJOA-10-2013-0716

[CIT0052] Vignoli, E., Prudhomme, N., Terriot, K., Cohen-Scali, V., Arnoux-Nicolas, C., Bernaud, J.L. et al., 2020, ‘Decent work in France: Context, conceptualization, and assessment’, *Journal of Vocational Behavior* 116(Part A), 103345. 10.1016/j.jvb.2019.103345

[CIT0053] Wang, I., Tsai, H., Lee, M. & Ko, R., 2021, ‘The effect of work–family conflict on emotional exhaustion and job performance among service workers: The cross-level moderating effects of organizational reward and caring’, *The International Journal of Human Resource Management* 32(14), 3112–3133. 10.1080/09585192.2019.1651373

[CIT0054] World Health Organization, 2020, *Guidelines on physical activity and sedentary behaviour*, World Health Organization, Geneva.

[CIT0055] Yang, F., Jiang, Y. & Pu, X., 2021, ‘Impact of work value perception on workers’ physical and mental health: Evidence from China’, *Healthcare (Switzerland)* 9(8), 1–21. 10.3390/healthcare9081059 PMC8393698 34442196

[CIT0056] Zondo, R.W.D., 2021, ‘Assessing the effectiveness of an occupational health and safety system in a selected automotive assembly organisation in South Africa’, *South African Journal of Economic and Management Sciences* 24(1), 1–8. 10.4102/sajems.v24i1.3553

[CIT0057] Zulfqar, A., Liaqat, A. & Nazir, A., 2012, ‘Organizational climate: A study of pharmaceutical industry in Pakistan’, *African Journal of Business Management* 6(49), 11880–11886. 10.5897/ajbm09.463

